# Identification of rickettsial isolates at the species level using multi-spacer typing

**DOI:** 10.1186/1471-2180-7-72

**Published:** 2007-07-30

**Authors:** Pierre-Edouard Fournier, Didier Raoult

**Affiliations:** 1 Unité des rickettsies, IFR 48, CNRS UMR 6020, Faculté de médecine, Université de la Méditerranée, 27 Boulevard Jean Moulin, 13385 Marseille cedex 05, France

## Abstract

**Background:**

In order to estimate whether multi-spacer typing (MST), based on the sequencing of variable intergenic spacers, could serve for the identification of *Rickettsia *at the species level, we applied it to 108 rickettsial isolates or arthropod amplicons that include representatives of 23 valid *Rickettsia *species.

**Results:**

MST combining the *dksA*-*xerC*, *mppA*-*purC*, and *rpmE*-tRNA^fMet ^spacer sequences identified 61 genotypes, allowing the differentiation of each species by at least one distinct genotype. In addition, MST was discriminatory at the strain level in six species for which several isolates or arthropod amplicons were available.

**Conclusion:**

MST proved to be a reproducible and high-resolution genotyping method allowing clear identification of rickettsial isolates at the species level and further additional differentiation of strains within some species.

## Background

*Rickettsiae *are obligate intracellular, Gram-negative bacteria, the vectors and reservoirs of which are mainly arthropods, although they may also infect vertebrate hosts [1]. Currently, the genus *Rickettsia *contains 24 validated species, 22 of which are classified into 2 groups: i) the typhus group including *R. prowazekii *[2] and *R. typhi *[3]; and ii)the spotted fever group made of *R. rickettsii *[4], *R. conorii *[5], *R. africae *[6], *R. sibirica *[7], *R. slovaca *[8], *R. honei *[9], *R. japonica *[10], *R. australis *[11], *R. akari *[12], *R. felis *[13], *R. aeschlimannii *[14], *R. helvetica *[15], *R. massiliae *[16], *R. rhipicephali *[17], *R. montanensis *[18], *R. parkeri *[19], *R. heilongjiangensis *[20], *R. tamurae *[21], *R. asiatica *[22], and *R. peacockii *[23]. Among spotted fever rickettsiae, *R. peacockii *is the only validated species for which no established strain is available. Two additional species, *i.e.*, *R. bellii *[24] and *R. canadensis *[25], initially classified within a third group named "ancestral group" [26], may not belong to a single group [20] but lie outside from the former two groups. In addition to the 24 recognized species, more than 20 rickettsial isolates which have not been fully characterized or have not received a species designation, have been described and their current classification is uncertain [27].

Various DNA-based techniques have been developed for the species identification of rickettsiae. Phylogenetic analysis based on the 16S rRNA gene sequences have frequently been used, but since the sequences are highly conserved, significant inferences about intragenus phylogeny are not possible [1,28]. In recent years, several genes have been sequenced for most of the known rickettsial isolates, including the citrate synthase – encoding gene (*glt*A) [29], and the *Rickettsia*-specific *ompA *[30], *ompB *[31], *sca*1 [32], *sca*2 [33], and *sca*4 [34] genes, encoding autotransporter proteins. The usefulness of DNA taxonomy has been recognized for living organisms [35] and Maiden *et al*. [36] have demonstrated the usefulness of multiple gene sequencing for prokaryotic taxonomy. Using multi-gene sequencing, we proposed gene sequence-based criteria for the identification of rickettsial isolates at the genus, group and species levels [20]. However, we observed that such a method lacks intra species discriminatory power [37]. As a consequence, there is no current method that both identifies rickettsiae at the species and strain levels, and produces results that are reproducible and comparable among laboratories.

Recently, we developed a new genotyping tool named Multi-Spacer Typing (MST), based on the assumption that intergenic spacers, which are less subject to evolutionary pressure than coding sequences, would be more appropriate for typing bacteria at the strain level than genes [37]. We demonstrated that MST using three intergenic spacers was suitable for typing *R. conorii *(31 genotypes among 42 tested strains), *R. sibirica *(3 genotypes among 14 tested strains), and *R. prowazekii *(4 genotypes among 15 studied strains) at the strain level, and produced species-specific signatures [37-39]. Herein, we hypothesized that the three intergenic spacers successfully used for *R. conorii*, being also present in *R. sibirica *and *R. prowazekii*, could also exhibit species-specific signatures in other *Rickettsia *species. Thus, in order to estimate the suitability of MST for identifying rickettsiae at the species level, we applied it to 20 additional species for which isolates are available [see Additional file 1].

## Results

### PCR amplification and sequencing

The *dksA*-*xerC*, *mppA*-*purC*, and *rpmE*-tRNA^fMet ^intergenic spacers were amplified from all the strains of *Rickettsia *studied except *R. canadensis *and *R. bellii*, for which the *mppA*-*purC *spacer PCR could not amplify any fragment.

**Figure 1:**
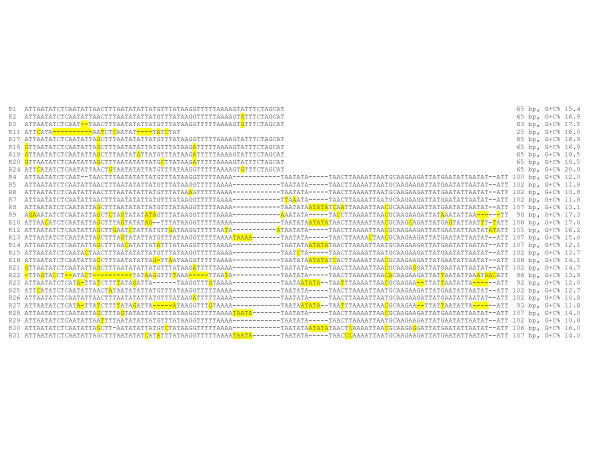
Repeated sequences making up the *dksA-xerC *intergenic spacers of *Rickettsia *species.

The size of the *dksA-xerC *spacer ranged from 92 for *R. prowazekii *to 687-bp for *R. montanensis*. Depending on species and strains, the *dksA-xerC *spacer was made of 31 different repeats named R1 to R31 that varied in size, from 25- to 107-bp, in number and in arrangement. The repeats were classified into two groups depending on their sizes, from 25- to 65-bp for the first group which includes 9 different repeats, and 86- to 107-bp for the second group. Sequences of both groups share a highly conserved 5'-part, and differ in their 3'-end. The sequences of these repeats are shown in Figure 1; and their repartition within the different *Rickettsia *strains reported in Additional file 2. Overall, the *dksA*-*xerC *spacer allowed the identification of 42 different genotypes. These included 19 genotypes within the *R. conorii *species, two within the *R. felis *species, three within the *R. sibirica *species, and two within the *R. slovaca *species. Other species each exhibited a unique *dksA*-*xerC *spacer sequence, except *R. africae *which had the same sequence as *R. conorii *subsp. *Caspia *isolate Chad, *R. parkeri *which had the same sequence as *R. conorii *subsp. *Caspia *isolate A-167, and *R. massiliae *which had the same sequence as *R. rhipicephali*.

*MppA*-*purC *amplicons ranged from 80-bp for *R. akari *to 180-bp for *R. felis*. The degree of pairwise nucleotide sequence similarity ranged from 50.5% between *R. conorii *and *R. felis *to 100% between *R. honei *and *R. africae *or *R. helvetica *and *R. tamurae*. Using the BLAST software, we identified, within *mppA-purC *spacer sequences, the presence of a 25- to 102-bp *Rickettsia *palindromic element, depending on species, that has a high degree of similarity with the *Rickettsia *palindromic element 1 (RPE-1) family [40,41]. Thirty-one distinct genotypes were identified among *mppA*-*purC *spacer sequences [see Additional file 2]. *R. conorii *strains were classified into eight genotypes, *R. felis *strains and amplicons into three genotypes, *R. massiliae *strains and amplicons into two genotypes, *R. sibrica *strains into three genotypes, and *R. slovaca *strain and amplicons into two genotypes. Each of the other species had a unique *mppA*-*purC *genotype, with the exception of *R. africae*, which exhibited the same genotype as *R. honei*, and *R. asiatica *and *R. tamurae *which exhibited the same genotype as *R. helvetica*.

The size of the *rpmE*-tRNA^fMet ^spacer sequence ranged from 175 for *R. typhi *to 402-bp for *R. asiatica*. The degree of pairwise nucleotide sequence similarity ranged from 69.6% between *R. typhi *and *R. aeschlimannii *to 100% between *R. honei *and *R. africae*. Using the BLAST software, we identified, within *rpmE*-tRNA^fMet ^spacer sequences, the presence of a *Rickettsia *palindromic element that has a high degree of similarity with the *Rickettsia *palindromic element 2 (RPE-2) family [40,41]. Twenty-nine distinct genotypes were identified among *rpmE*-tRNA^fMet ^spacer sequences [see Additional file 2]. *R. conorii *strains were classified into four genotypes, *R*. *felis *strain and amplicons into two genotypes, *R. prowazekii *strains and amplicons into three genotypes, and *R. sibirica *strains into two genotypes. Each of the other species exhibited a unique *rpmE*-tRNA^fMet ^spacer sequence, except *R. africae *and *R. honei*, which had the same sequence.

### MST genotyping

By combining the genotypes obtained from the three intergenic spacers we studied, we identified 61 MST genotypes among the 108 strains and arthropod amplicons. Thirty-one MST genotypes were identified within the *R. conorii *species, three within *R. felis*, two within *R. massiliae*, three within *R. prowazekii*, three within *R. sibirica*, and three within *R. slovaca*. Each other studied species had a unique MST genotype. Each *Rickettsia *species was identified on the basis of a specific *dksA-xerC *sequence except for *R. africae*, *R. conorii *subsp. *Caspia*, *R. massiliae*, *R. parkeri *and *R. rhipicephali *for which the three spacer sequences were necessary.

## Discussion

The ability to differentiate bacteria beyond the species level is essential for identifying and tracking infectious disease outbreaks and to improve our knowledge of the population genetics, epidemiology, and ecology of bacterial pathogens. In the past decades, a variety of phenotypic and genotypic methods have been used for microbial typing, identification, and classification. Commonly used subtyping methods, such as serotyping, phage typing, ribotyping, and pulsed-field gel electrophoresis, are time-consuming. PCR and DNA sequence-based typing methods have emerged as the most rapid, reliable, and simple ways to characterize and type microorganisms and can reduce interpretation ambiguity. To be reliable, a typing method should not be influenced by sequence variations that would occur during culture passage.

*Rickettsia *species, as intracellular bacteria, undergo lower rates of genomic changes than extracellular bacteria, and their genomes, with the exception of *R. bellii *[42], are highly collinear [42-46]. As a consequence, intergenic spacers, previously demonstrated to be more variable than coding sequences [37], are a valuable target for genotyping these bacteria. The most widely used intergenic spacer in bacteria, *i. e.*, 16S–23S rRNA, is not useable in *Rickettsia *species as both genes are not contiguous [44]. We have previously demonstrated the versatility of MST by applying it to several bacterial species in addition to rickettsiae, including *Yersinia pestis *[47], *Coxiella burnetii *[48], *Bartonella quintana *[49] and *Bartonella henselae *[50]. For rickettsiae, we have demonstrated that it is insensitive to culture passage in both *R. conorii *and *R. prowazekii *[37,38]. This makes MST a reliable typing method for strain tracing. The three intergenic spacers used in our study were previously selected among the most variable intergenic spacers conserved in both the *R. conorii *and *R. prowazekii *genomes [37]. We could detect the three spacers in all studied rickettsial strains and arthropod amplicons except for the *mppA-purC *spacer that could not be found in *R. canadensis *and *R. bellii*. This was explained in the latter species by the fact that the *mppA *and *purC *genes are not contiguous in the genome [42]. To date, only one gene, *i. e.*, *sca*1, a member of the *sca*-family of auto-transporter genes, has been found in all *Rickettsia *species [32]. However, this gene was shown to be discriminatory at the species level but not at the intra-species level. In contrast, MST identified 61 distinct genotypes that include genotypes specific of each of the 23 studied species. To the best of our knowledge, this is the first reproducible method that allows identification of rickettsiae at the species and strain levels and can be applied to both isolates and clinical or arthropod specimens. In addition to the previously studied *R. conorii*, *R. prowazekii *and *R. sibirica *species for which MST has demonstrated intra-species variability, we showed that this method was also discriminatory for *R. felis*, *R. massiliae*, and *R. slovaca *strains and amplicons. Therefore, the discriminatory power of MST was highly correlated to the analyzed species. For *R. africae*, for which a large number of strains and tick amplicons from various geographic origins were tested, this lower genetic diversity may reflect a more recent emergence of this species than *R. conorii*. However, we cannot exclude that the observed differences could be due to a bias in specimen selection.

We identified repeated elements within the three studied spacers. The high density of repeated sequences appears to be a characteristic of *Rickettsia *genomes [40,41,45]. These repeated sequences are classified into several categories. The most remarkable of these elements are the RPEs, which represent 3.2% of the *R. conorii *genome [41]. The RPEs identified within the *mppA-purC *and *rpmE*-tRNA^fMet ^spacers belong to the RPE-1 and RPE-2 families, respectively. Although usually confined to intergenic regions in prokaryotes [51], some RPE families, including the RPE-1 and RPE-2 families, are also found inserted in-frame within genes in *Rickettsia *genomes [41,45]. Their exact function at that location is unknown but it has been suggested that they may play a role in the creation and the modification of proteins [41]. The presence of RPEs in the studied intergenic spacers may in part explain their high rate of interspecies nucleotide variability.

Within the third intergenic spacer, *i.e.*, *dksA-xerC*, we have previously identified a second category of repeated elements named variable number of tandem repeats (VNTRs). These repeats are present at a single genomic locus and show interindividual length variability [51]. Such VNTRs are recognized in eukaryotes as source of DNA variability [52], and are also described in bacteria, where they are found within genes and mainly play a role in implementing size variation of membrane-associated proteins. Such a location is found within the *ompA *gene in rickettsiae. The main characteristic of the VNTRs herein described is their unusual presence within a non-coding sequence. We identified 31 repeat types, classified into two groups depending on their sizes. Another feature of these VNTRs is that their number and arrangement vary depending on strains and species, from a single repeat to 10 repeats. Such a variability makes the *dksA-xerC *the most discriminatory of the three spacers, with 42 genotypes identified. In a recent work, Vitorino *et al. *proposed that PCR amplification of VNTRs within the *dksA-xerC *might serve to identify rickettsial isolates [53]. However, these authors only took into account the size of the VNTRs, not their sequence variability. Using optimal conditions of electrophoresis resolution that would discriminate size differences of 1-bp, which may be difficult to achieve in routine laboratories, PCR amplification of VNTRs would discriminate only 22 different types among our 42 *dksA-xerC *genotypes. As an example, this would not differentiate *R. felis *from *R. massiliae *and *R. helvetica*, all three being recognized or suspected human pathogens. This supports the MST method that relies on DNA sequences.

## Conclusion

MST using the *dks*A-*xer*C, *mpp*A-*pur*C, and *rpm*E-tRNA^fMet ^intergenic spacers is an efficient and reproducible typing method able to identify 23 validated *Rickettsia *species. It also allows the discrimination at the strain level in six species. Thus, it constitutes a powerful tool for identification of these bacteria, in particular within clinical specimens.

## Methods

### Rickettsial strains or PCR amplicons

The strains or PCR amplicons used in this study are listed in Additional file 1. Rickettsial strains were propagated at 32°C on Vero cell (ATCC CRL-1587) monolayers in Eagle's minimal essential medium (MEM, Seromed, Berlin, Germany) supplemented with 4% fetal bovine serum (Seromed) and 2 mM glutamine. When cells stained with Gimenez were heavily infected (3 to 5 days), the cultures were harvested, centrifuged (12,000 × *g *for 10 min), resuspended in MEM and stored at -70°C until processed further.

For the three *Rickettsia *species previously studied by MST, we included a representative strain of each distinct genotype, *i.e.*, 27 strains for *R. conorii *subsp. *conorii *[37], two for *R. conorii *subsp. *caspia*, and one each for *R. conorii *subsp. *indica *and *R. conorii *subsp. *israelensis *[54]; one strain for *R. sibirica *subsp. *sibirica*, and two for *R. sibirica *subsp. *mongolitimonae *[39]; and two strains and a louse amplicon for *R. prowazekii *[38].

For the other 20 valid *Rickettsia *species studied, we included a minimum of one strain and, when available, several strains and/or arthropod amplicons [see Additional file 1]. These extra-strains or arthropod amplicons had been identified using *gltA *and *ompB *gene amplification and sequencing as previously described [28,29]. All studied strains are detailed in Additional file 1.

### PCR amplification and sequencing

Genomic DNA was extracted from rickettsial cultures using the QIAamp Tissue kit (QIAGEN, Hilden, Germany) according to the manufacturer's instructions. We used the primers *dksA*F: 5'-TCCCATAGGTAATTTAGGTGTTTC-3' and *dksA*R: 5'-TACTACCGCATATCCAATTAAAAA-3', *mppA*F: 5'-GCAATTATCGGTCCGAATG-3' and *mppA*R: 5'-TTTCATTTATTTGTCTCAAAATTCA-3', and *rpmE*F: 5'-TTCCGGAAATGTAGTAAATCAATC-3' and *rpmE*R: 5'-TCAGGTTATGAGCCTGACGA-3', to amplify the intergenic spacers *dksA-xerC*, *mppA-purC*, and *rpmE*-tRNA^fMet^, respectively. The primers were obtained from Eurogentec (Seraing, Belgium). PCR reactions were carried out in a PTC-200 automated thermal-cycler (MJ Research, Waltham, Mass.) using the previously described conditions [37]. PCR products were purified using a QIAquick Spin PCR purification kit (QIAGEN) as described by the manufacturer. Sequencing reactions were carried out using the d-Rhodamine Terminator cycle sequencing ready reaction kit with Amplitaq Polymerase FS (Applied Biosystems, Coignieres, France) as described by the manufacturer. For all PCR products, sequences from both DNA strands were determined twice. Sequencing products were resolved using an ABI 3100 automated sequencer (Applied Biosystems). Sequence analysis was performed using the software package ABI Prism DNA Sequencing Analysis Software version 3.0 (Applied Biosystems). Sterile water was used as a negative control in each assay.

Sequences were deposited in GenBank under the accession numbers detailed in Additional file 1.

## Authors' contributions

The individual parts of the work presented in the paper were conducted as follows: PEF participated in the design of the study, carried out the molecular genetic studies, analyzed the sequences and drafted the manuscript. DR conceived the study and helped to draft the manuscript. Both authors read and approved the final manuscript.

## Supplementary Material

Additional file 1 *Rickettsia *strains studied. The Table details the strains studied and their GenBank accession numbers. Click here for file

Additional file 2 Genotypes obtained from each *Rickettsia *strain studied. The Table contains all genotypes obtained from the *Rickettsia *strains studied. Click here for file
