# Supplementary material for: Identification of rickettsial isolates at the species level using multi-spacer typing
Source: BMC Microbiol. 2007 Jul 30;7:72. doi: 10.1186/1471-2180-7-72 (PMC1950309; doi:10.1186/1471-2180-7-72)
Supplement: Additional file 1 — Rickettsia strains studied. The Table details the strains studied and their GenBank accession numbers. [file 1471-2180-7-72-S1.doc]

**Additional file 1:** *Rickettsia* strains studied

| **Species name** | **Strain**  **(ATCC number)** | **Strain number** | **Geographical origin** | GenBank accession number | | |
| --- | --- | --- | --- | --- | --- | --- |
|  | **or athropod amplicon** |  |  | *dksA*-*xerC* | *mppA*-*pur*C | *rpmE*-tRNA^fMet^ |
| *R. aeschlimannii* | MC16 * | 1 | Morocco | DQ008272 | DQ008293 | DQ008255 |
|  | 26438 ¶ | 2 | Algeria | DQ008272 | DQ008293 | DQ008255 |
|  | 26440 ¶ | 3 | Algeria | DQ008272 | DQ008293 | DQ008255 |
|  | 26443 ¶ | 4 | Algeria | DQ008272 | DQ008293 | DQ008255 |
|  | 26446 ¶ | 5 | Algeria | DQ008272 | DQ008293 | DQ008255 |
|  | 26447 ¶ | 6 | Algeria | DQ008272 | DQ008293 | DQ008255 |
| *R. africae* | ESF-5 * | 7 | Ethiopia | DQ008280 | DQ008301 | DQ008246 |
|  | URRCRAF21 * | 8 | South Africa | DQ008280 | DQ008301 | DQ008246 |
|  | URRCRAF23 * | 9 | South Africa | DQ008280 | DQ008301 | DQ008246 |
|  | URRCRAF24 * | 10 | South Africa | DQ008280 | DQ008301 | DQ008246 |
|  | URRCRAF26 * | 11 | South Africa | DQ008280 | DQ008301 | DQ008246 |
|  | URRCRAF34 * | 12 | South Africa | DQ008280 | DQ008301 | DQ008246 |
|  | URRCRAF42 * | 13 | South Africa | DQ008280 | DQ008301 | DQ008246 |
|  | URRCRAF63 * | 14 | South Africa | DQ008280 | DQ008301 | DQ008246 |
|  | URRCRAF84 * | 15 | South Africa | DQ008280 | DQ008301 | DQ008246 |
|  | URRCRAF87 * | 16 | South Africa | DQ008280 | DQ008301 | DQ008246 |
|  | URRCRAF89 * | 17 | South Africa | DQ008280 | DQ008301 | DQ008246 |
|  | URRCRAF90 * | 18 | South Africa | DQ008280 | DQ008301 | DQ008246 |
|  | URRCRAF93 * | 19 | South Africa | DQ008280 | DQ008301 | DQ008246 |
|  | 19343 ¶ | 20 | Reunion | DQ008280 | DQ008301 | DQ008246 |
|  | 19348 ¶ | 21 | Reunion | DQ008280 | DQ008301 | DQ008246 |
|  | 19740 ¶ | 22 | Martinique | DQ008280 | DQ008301 | DQ008246 |
|  | 19741 ¶ | 23 | Martinique | DQ008280 | DQ008301 | DQ008246 |
|  | 19742 ¶ | 24 | Martinique | DQ008280 | DQ008301 | DQ008246 |
|  | 19743 ¶ | 25 | Martinique | DQ008280 | DQ008301 | DQ008246 |
|  | 19744 ¶ | 26 | Martinique | DQ008280 | DQ008301 | DQ008246 |
|  | 19746 ¶ | 27 | Martinique | DQ008280 | DQ008301 | DQ008246 |
|  | 19267 ¶ | 28 | Saint Kitts and Nevis | DQ008280 | DQ008301 | DQ008246 |
|  | 19275 ¶ | 29 | Saint Kitts and Nevis | DQ008280 | DQ008301 | DQ008246 |
|  | 19300 ¶ | 30 | Saint Kitts and Nevis | DQ008280 | DQ008301 | DQ008246 |
|  | 19301 ¶ | 31 | Saint Kitts and Nevis | DQ008280 | DQ008301 | DQ008246 |
|  | 19305 ¶ | 32 | Saint Kitts and Nevis | DQ008280 | DQ008301 | DQ008246 |
|  | 19328 ¶ | 33 | Saint Kitts and Nevis | DQ008280 | DQ008301 | DQ008246 |
| *R. akari* | MK (VR-148) * | 34 | USA | DQ008270 | DQ008291 | DQ008260 |
| *R. asiatica* | IO-1 (CSUR R2^T^) * | 35 | Japan | EF123265 | DQ008296 | AF123269 |
| *R. australis* | Phillips * | 36 | Australia | DQ008273 | DQ008294 | DQ008259 |
| *R. bellii* | 369L42-1 * | 37 | USA | DQ008282 | ** | DQ852358 |
| *R. canadensis* | 2678 * | 38 | Canada | DQ008274 | ** | DQ852359 |
| *R. conorii* subsp. *conorii* | URRCCroatia29 * | 39 | Croatia | AY428738 | AY345087 | AY345092 |
|  | 16B * | 40 | Spain | AY428739 | AY345087 | AY345091 |
|  | M1 * | 41 | Georgia | AY428739 | AY345087 | AY345092 |
|  | Portugal4S * | 42 | Portugal | AY428739 | AY345085 | AY345091 |
|  | URRCFranceFEe8 * | 43 | France | AY428739 | AY345085 | AY345092 |
|  | URRCFranceFE11 * | 44 | France | AY428739 | AY345089 | AY345092 |
|  | URRCFranceFE17 * | 45 | France | AY428740 | AY345089 | AY345092 |
|  | URRCFranceFEe48 * | 46 | France | AY428740 | AY465118 | AY345092 |
|  | URRCTurkey58 * | 47 | Turkey | AY428740 | AY345087 | AY345091 |
|  | URRCFrance1 * | 48 | France | AY428741 | AY345089 | AY345092 |
|  | URRCFranceFEe4 * | 49 | France | AY428741 | AY345087 | AY345092 |
|  | URRCFranceFEe57 * | 50 | France | AY428741 | AY345089 | AY345091 |
|  | Zim1 * | 51 | Zimbabwe | AY428742 | AY345087 | AY345092 |
|  | ZimA * | 52 | Zimbabwe | AY428742 | AY345086 | AY345092 |
|  | URRCFranceFEe49 * | 53 | France | AY428743 | AY345089 | AY345092 |
|  | Malish, seven  (VR-613) * | 54 | South Africa | AY428744 | AY345087 | AY345092 |
|  | Kenya * | 55 | Kenya | AY428745 | AY345087 | AY345092 |
|  | URRCSpain3 * | 56 | Spain | AY428746 | AY345089 | AY345092 |
|  | Portugal1454 * | 57 | Portugal | AY428746 | AY345087 | AY345091 |
|  | SV9 * | 58 | Spain | AY428746 | AY345087 | AY345092 |
|  | URRCFranceFEe6 * | 59 | France | AY428747 | AY345089 | AY345092 |
|  | URRCFranceFEe2 * | 60 | France | AY428748 | AY345089 | AY345092 |
|  | Portugal821 * | 61 | Portugal | AY428749 | AY345087 | AY345091 |
|  | Moroccan (VR-141) * | 62 | Morocco | AY428750 | AY345087 | AY345092 |
|  | URRCTurkey59 * | 63 | Turkey | AY462116 | AY345087 | AY345091 |
|  | URRCTurkey61 * | 64 | Turkey | AY497559 | AY345087 | AY345091 |
| *R. conorii* subsp. *indica* | ITTR (VR-597) * | 65 | India | AY836513 | AY836517 | AY345092 |
| *R. conorii* subsp. *Israelensis* | ISTTCDC1 * | 66 | Israel | AY836510 | AY836514 | AY836518 |
| *R. conorii* subsp. *caspia* | A-167 * | 67 | Russia | AY836511 | AY836516 | AY836519 |
|  | Chad * | 68 | Chad | AY836512 | AY836516 | AY836519 |
| *R. felis* | URRWXCal2 (VR-1525) * | 69 | USA | DQ008281 | DQ008302 | DQ008258 |
|  | 26457 † | 70 | Algeria | DQ648584 | DQ648589 | DQ648585 |
|  | 26460 † | 71 | Algeria | DQ648584 | DQ648589 | DQ648585 |
|  | 2460634 † | 72 | New Zealand | DQ008281 | DQ648590 | DQ008258 |
|  | 2460702 † | 73 | New Zealand | DQ008281 | DQ648590 | DQ008258 |
|  | 2460706 † | 74 | New Zealand | DQ008281 | DQ648590 | DQ008258 |
| *R. heilongjiangensis* | 054 (CSUR R^T^) * | 75 | China | EF123264 | EF123267 | EF123268 |
| *R. helvetica* | C9P9 * | 76 | Switzerland | DQ008275 | DQ008296 | DQ648586 |
|  | IP-1 * | 77 | Japan | DQ008275 | DQ008296 | DQ648586 |
|  | IM-1 * | 78 | Japan | DQ008275 | DQ008296 | DQ648586 |
|  | Tick21 ¶ | 79 | France | DQ008275 | DQ008296 | DQ648586 |
|  | Tick35 ¶ | 80 | France | DQ008275 | DQ008296 | DQ648586 |
| *R. honei* | TT-118 (VR-599) * | 81 | Thailand | DQ008276 | DQ008301 | DQ008252 |
| *R. japonica* | YM * | 82 | Japan | DQ008277 | DQ008298 | DQ008253 |
| *R. massiliae* | Mtu1 * | 83 | France | DQ008267 | DQ008288 | DQ008256 |
|  | Bar29 * | 84 | Spain | DQ008267 | DQ648587 | DQ008256 |
|  | 2562683 ¶ | 85 | Italy | DQ008267 | DQ648587 | DQ008256 |
|  | 2562702 ¶ | 86 | Italy | DQ008267 | DQ648587 | DQ008256 |
|  | 2562703 ¶ | 87 | Italy | DQ008267 | DQ648587 | DQ008256 |
|  | 2562704 ¶ | 88 | Italy | DQ008267 | DQ648587 | DQ008256 |
|  | 2562706 ¶ | 89 | Italy | DQ008267 | DQ648587 | DQ008256 |
| *R. montanensis* | M/5-6 * | 90 | USA | DQ008279 | DQ008300 | DQ008254 |
| *R. parkeri* | Maculatum 20 * | 91 | USA | DQ008266 | DQ008287 | DQ008249 |
|  | Portsmouth * | 92 | USA | DQ008266 | DQ008287 | DQ008249 |
| *R. prowazekii* | Breinl (VR-142) * | 93 | Poland | DQ008269 | DQ008290 | AY695448 |
|  | Madrid E * | 94 | Spain | DQ008269 | DQ008290 | AY695447 |
|  | Bur12749‡ | 95 | Burundi | DQ008269 | DQ008290 | AY695449 |
| *R. rhipicephali* | 3-7-6 * | 96 | USA | DQ008268 | DQ008289 | DQ008257 |
| *R. rickettsii* | R, Bitteroot  (VR-891) * | 97 | USA | DQ008265 | DQ008286 | DQ008250 |
| *R. sibirica* subsp. sibirica | 246 (VR-151) * | 98 | Russia | DQ008262 | DQ008283 | DQ008247 |
| *R. sibirica* subsp. *mongolitimonae* | HA-91 (VR-1526) * | 99 | China | DQ008263 | DQ008284 | DQ008248 |
|  | URRMTMFEe65 * | 100 | Algeria | DQ008264 | DQ008285 | DQ008248 |
| *R. slovaca* | 13-B * | 101 | Slovakia | DQ008278 | DQ008299 | DQ008251 |
|  | 2460360 ¶ | 102 | France | DQ008278 | DQ008299 | DQ008251 |
|  | 2562452 ¶ | 103 | France | DQ008278 | DQ008299 | DQ008251 |
|  | 2461180 ¶ | 104 | France | DQ648583 | DQ008299 | DQ008251 |
|  | 2464783 ¶ | 105 | France | DQ648583 | DQ008299 | DQ008251 |
|  | 2564130 ¶ | 106 | France | DQ648583 | DQ648588 | DQ008251 |
| *R. tamurae* | AT-1 (CSUR R1^T^) | 107 | Japan | EF123266 | DQ008296 | EF123270 |
| *R. typhi* | Wilmington (VR-144) * | 108 | USA | DQ008271 | DQ008292 | DQ008261 |

* = rickettsial isolate; ¶ = tick amplicons; † = flea amplicons; ‡ = body louse amplicon; ** = spacer does not exist.
