# Supplementary material for: Identification of rickettsial isolates at the species level using multi-spacer typing
Source: BMC Microbiol. 2007 Jul 30;7:72. doi: 10.1186/1471-2180-7-72 (PMC1950309; doi:10.1186/1471-2180-7-72)
Supplement: Additional file 2 — Genotypes obtained from each Rickettsia strain studied. The Table contains all genotypes obtained from the Rickettsia strains studied. [file 1471-2180-7-72-S2.doc]

**Additional file 2:** Genotypes obtained from each *Rickettsia* strain studied.

| ***Rickettsia* species** | **Strain number*** | ***dksA-xerC*** | | |  | ***mppA-purC*** | |  | ***rpmE-*tRNA^fMe^** | | **MST**  **Genotype** |
| --- | --- | --- | --- | --- | --- | --- | --- | --- | --- | --- | --- |
|  |  | **Genotype number** | **Size (bp)** | **VNTR** |  | **Genotype number** | **Size (bp)** |  | **Genotype number** | **Size (bp)** |  |
| *R. aeschlimannii* | 1 – 6 | W | 107 | R8 |  | AD | 146 |  | J | 339 | 37 |
| *R. africae* | 7 – 33 | S | 167 | R2R5 |  | M | 153 |  | K | 269 | 38 |
| *R. akari* | 34 | X | 98 | R9 |  | N | 80 |  | L | 290 | 39 |
| *R. asiatica* | 35 | AN | 107 | R31 |  | Q | 113 |  | AA | 402 | 59 |
| *R. australis* | 36 | Y | 106 | R10 |  | O | 139 |  | M | 285 | 40 |
| *R. bellii* | 37 | Z | 111 | R11R22 |  |  | No¶ |  | N | 385 | 41 |
| *R. canadensis* | 38 | AA | 105 | R12 |  |  | No¶ |  | O | 345 | 42 |
| *R. conorii* subsp. *conorii* | 39 | A | 549 | R1R1R2R3R3R2R3R4 |  | B | 153 |  | B | 268 | 1 |
|  | 40 | B | 549 | R1R2R3R3R3R2R3R5 |  | B | 153 |  | A | 269 | 2 |
|  | 41 | B | 549 | R1R2R3R3R3R2R3R5 |  | B | 153 |  | B | 268 | 3 |
|  | 42 | B | 549 | R1R2R3R3R3R2R3R5 |  | D | 153 |  | A | 269 | 4 |
|  | 43 | B | 549 | R1R2R3R3R3R2R3R5 |  | D | 153 |  | B | 268 | 5 |
|  | 44 | B | 549 | R1R2R3R3R3R2R3R5 |  | A | 153 |  | B | 268 | 6 |
|  | 45 | C | 484 | R2R3R3R3R2R3R5 |  | A | 153 |  | B | 268 | 7 |
|  | 46 | C | 484 | R2R3R3R3R2R3R5 |  | E | 153 |  | B | 268 | 8 |
|  | 47 | C | 484 | R2R3R3R3R2R3R5 |  | B | 153 |  | A | 269 | 9 |
|  | 48 | D | 486 | R1R2R3R3R3R2R5 |  | A | 153 |  | B | 268 | 10 |
|  | 49 | D | 486 | R1R2R3R3R3R2R5 |  | B | 153 |  | B | 268 | 11 |
|  | 50 | D | 486 | R1R2R3R3R3R2R5 |  | A | 153 |  | A | 269 | 12 |
|  | 51 | E | 423 | R1R1R2R2R3R4 |  | B | 153 |  | B | 268 | 13 |
|  | 52 | E | 423 | R1R1R2R2R3R4 |  | C | 153 |  | B | 268 | 14 |
|  | 53 | F | 421 | R2R3R3R3R2R5 |  | A | 153 |  | B | 268 | 15 |
|  | 54 | G | 358 | R1R2R2R3R4 |  | B | 153 |  | B | 268 | 16 |
|  | 55 | H | 360 | R1R1R1 R2R4 |  | B | 153 |  | B | 268 | 17 |
|  | 56 | I | 356 | R3R3R2R3R5 |  | A | 153 |  | B | 268 | 18 |
|  | 57 | I | 356 | R3R3R2R3R5 |  | B | 153 |  | A | 269 | 19 |
|  | 58 | I | 356 | R3R3R2R3R5 |  | B | 153 |  | B | 268 | 20 |
|  | 59 | J | 356 | R3R3R3R2R5 |  | A | 153 |  | B | 268 | 21 |
|  | 60 | K | 360 | R2R2R3R2R5 |  | A | 153 |  | B | 268 | 22 |
|  | 61 | L | 228 | R2R3R4R2R5 |  | B | 153 |  | A | 269 | 23 |
|  | 62 | M | 100 | R4 |  | B | 153 |  | B | 268 | 24 |
|  | 63 | N | 486 | R2R3R3R3R2R2R5 |  | B | 153 |  | A | 269 | 25 |
|  | 64 | O | 484 | R1R2R2R3R3R2R4 |  | B | 153 |  | A | 269 | 26 |
| *R. conorii* subsp. *Indica* | 65 | P | 165 | R2R4 |  | F | 153 |  | B | 268 | 27 |
| *R. conorii* subsp. *Israelensis* | 66 | Q | 232 | R1R2R5 |  | G | 153 |  | C | 269 | 28 |
| *R. conorii* subsp. *Caspia* | 67 | R | 232 | R2R2R5 |  | H | 153 |  | D | 269 | 29 |
|  | 68 | S | 167 | R2R5 |  | H | 153 |  | D | 269 | 30 |
| *R. felis* | 69 | AB | 107 | R13 |  | P | 180 |  | P | 222 | 43 |
|  | 70, 71 | AM | 107 | R28 |  | AB | 156 |  | Z | 222 | 57 |
|  | 72 – 74 | AB | 107 | R13 |  | AC | 181 |  | P | 222 | 58 |
| *R. heilongjiangensis* | 75 | AO | 167 | R2R29 |  | AF | 153 |  | AB | 291 | 60 |
| *R. helvetica* | 76 – 80 | AC | 107 | R14 |  | Q | 113 |  | Q | 334 | 44 |
| *R. honei* | 81 | AJ | 297 | R2R2R2R5 |  | M | 153 |  | K | 269 | 45 |
| *R. japonica* | 82 | AD | 167 | R2R15 |  | R | 153 |  | R | 291 | 46 |
| *R. massiliae* | 83 | AE | 106 | R16 |  | S | 152 |  | S | 293 | 47 |
|  | 84 – 89 | AE | 106 | R16 |  | Z | 152 |  | S | 293 | 48 |
| *R. montanensis* | 90 | AF | 687 | R17R18R17R17R18R18R18R19R20R21 |  | T | 160 |  | T | 333 | 49 |
| *R. parkeri* | 91, 92 | R | 232 | R2R2R5 |  | U | 153 |  | U | 209 | 50 |
| *R. prowazekii* | 93 | AG | 92 | R23 |  | I | 133 |  | E | 186 | 31 |
|  | 94 | AG | 92 | R23 |  | I | 133 |  | F | 186 | 32 |
|  | 95 | AG | 92 | R23 |  | I | 133 |  | G | 267 | 33 |
| *R. rhipicephali* | 96 | AE | 106 | R16 |  | V | 153 |  | V | 332 | 51 |
| *R. rickettsii* | 97 | AH | 167 | R24R25 |  | W | 153 |  | W | 269 | 52 |
| *R. sibirica* subsp. *sibirica* | 98 | T | 102 | R6 |  | J | 153 |  | H | 269 | 34 |
| *R. sibirica* subsp. *mongolitimonae* | 99 | U | 102 | R5 |  | K | 153 |  | I | 269 | 35 |
|  | 100 | V | 102 | R7 |  | L | 153 |  | I | 269 | 36 |
| *R. slovaca* | 101 – 103 | AK | 232 | R2R2R26 |  | X | 151 |  | X | 269 | 53 |
|  | 104, 105 | AL | 167 | R2R26 |  | X | 151 |  | X | 269 | 55 |
|  | 106 | AL | 167 | R2R26 |  | AA | 151 |  | X | 269 | 56 |
| *R. tamurae* | 107 | AP | 106 | R30 |  | Q | 113 |  | AC | 292 | 61 |
| *R. typhi* | 108 | AI | 93 | R27 |  | Y | 121 |  | Y | 175 | 54 |

*Strain number refers to numbering of Table 1; ¶No = spacer does not exist
